# Correlation between posterior tibial slope and sagittal alignment under weight-bearing conditions in osteoarthritic knees

**DOI:** 10.1371/journal.pone.0202488

**Published:** 2018-09-12

**Authors:** Tomoharu Mochizuki, Osamu Tanifuji, Yoshio Koga, Takashi Sato, Koichi Kobayashi, Satoshi Watanabe, Toshihide Fujii, Hiroshi Yamagiwa, Ryota Katsumi, Hiroshi Koga, Go Omori, Naoto Endo

**Affiliations:** 1 Division of Orthopedic Surgery, Department of Regenerative and Transplant Medicine, Niigata University Graduate School of Medical and Dental Science, Niigata, Japan; 2 Department of Orthopaedic Surgery, Nioji Onsen Hospital, Niigata, Japan; 3 Department of Orthopaedic Surgery, Niigata Medical Center, Niigata, Japan; 4 Department of Health Sciences, Niigata University School of Medicine, Niigata, Japan; 5 Department of Orthopaedic Surgery, Saiseikai Niigata Daini Hospital, Niigata, Japan; 6 Department of Health and Sports, Faculty of Health Sciences, Niigata University of Health and Welfare, Niigata, Japan; Consorci Parc de Salut MAR de Barcelona, SPAIN

## Abstract

**Introduction:**

Posterior tibial slope (PTS) and sagittal alignment are important factors in the etiology of knee osteoarthritis and knee surgery. Clinically, sagittal alignment, which indicates flexion contracture of the knee, contributes to knee function in weight-bearing (WB) conditions. PTS and sagittal alignment under WB conditions in varus osteoarthritic knees are presumed to affect each other, but their association remains unclear. In this study, we aimed to clarify the association.

**Material and methods:**

In total, 140 osteoarthritic varus knees were investigated. Under WB conditions, a three-dimensional (3D) alignment assessment system was applied via biplanar long-leg X-rays, using 3D-to-2D image registration technique. The evaluation parameters were as follows: 1) 3D mechanical flexion angle (3DMFA) in regards to sagittal alignment, 2) passing point in the WB line (PP), and 3) medial and lateral PTS.

**Results:**

The medial and lateral PTS showed a positive correlation with 3DMFA and PP, respectively (medial PTS–3DMFA, *p* = 0.001; medial PTS–PP, *p* < 0.0001; lateral PTS–3DMFA, *p* < 0.0001; lateral PTS–PP, *p* = 0.002). The flexion contracture group with 3DMFA >5° demonstrated greater PTS than non-flexion contracture group (medial PTS, *p* = 0.006; lateral PTS, *p* = 0.006).

**Conclusions:**

Both medial and lateral PTS were correlated with sagittal alignment under WB conditions and were larger in the flexion contracture group. This finding can explain the function to take the load articular surface parallel to the ground for holding the balance in WB conditions in the sagittal plane for osteoarthritic knees. Moreover, surgeons may be required to decrease the PTS during knee arthroplasty to restore full extension in knees of patients with fixed flexion contracture.

## Introduction

**Fig 1:**
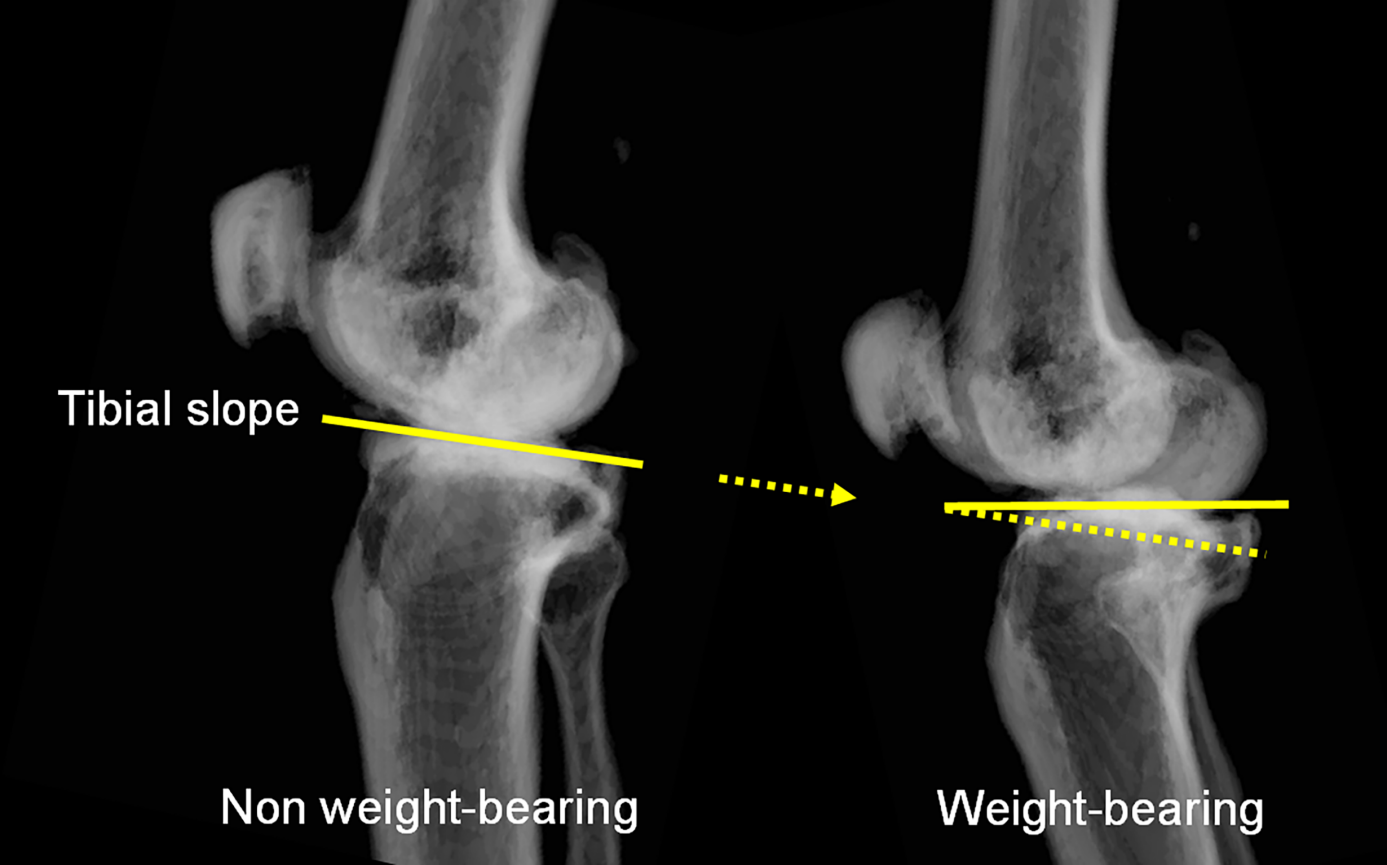
Schematic diagram showing that the articular surface is aligned parallel to the ground under WB conditions in order to efficiently take WB load in the standing position.

The specific human faculty of vertical balance synchronizes any action with a skeletomuscular adjustment [1]. To achieve the complicated motions of human knees, functions corresponding to the shapes and motions of the load joints evolved. The proximal articular surface of the tibia holds the distal articular surface of the femur and assumingly functions as a load bearing horizontal surface to balance and bipedal movement under weight-bearing (WB) conditions. In osteoarthritic knees, it has been reported that an incline in the coronal plane of the tibial medial compartment of the proximal articular surface is aligned parallel to the ground under WB conditions (parallel mechanism) [2]. As such, the parallel mechanism is also assumed to function in WB conditions in the sagittal plane of the tibial articular surface. For example, subjects with an increased posterior tibial slope (PTS) may take the standing position using more knee flexion (sagittal alignment) under WB conditions in order to align the proximal tibial articular surface parallel to the ground (Fig 1). A parallel mechanism of the tibial articular surface in the sagittal plane would be indirectly evidenced by a PTS and sagittal alignment correlation under WB conditions.

PTS strongly influences the outcome and function in knee arthroplasty [3–7]. PTS is a crucial factor that affects anteroposterior stability [4, 8, 9], range of motion, tension on crucial knee ligaments, and the balance of flexion and extension gaps [3, 6] in knee arthroplasty. Moreover, PTS plays a key biomechanical role in knee kinematics. A greater PTS increases the shear force and anterior translation of the tibia to the femur, resulting in a more posterior translation of the tibiofemoral contact point [4, 5, 7]. If PTS was correlated with sagittal alignment of an angle nearly equal to flexion contracture of the knee, the subjects with larger flexion contracture in the preoperative condition would have the greater PTS. For these patients, surgeons should exercise care in determining the PTS angle for knees with flexion contracture prior to knee surgery.

To evaluate sagittal alignment in WB conditions and PTS in medial and lateral compartments in a three-dimensional (3D) space, a special 3D assessment system for alignment and morphology is necessary. Common modalities, such as a plain radiography and computed tomography (CT), cannot assess 3D alignment under WB conditions. In addition, to accurately assess the morphology and alignment parameters, a coordinate system and automatic calculation, are needed. In our group, the 3D lower extremity alignment assessment system (Knee CAS; LEXI, Inc., Tokyo, Japan) for use under WB conditions was developed [10–24]. Within this system, the 3D digital bone models, incorporating the coordinate system and information related to the parameters of morphology and alignment, are projected on biplanar radiographies using a 3D-to-2D image registration technique [10–24]. As a result, the system can automatically evaluate all variables, including whole lower leg alignment and bone morphology under WB conditions with high accuracy [11].

The purpose of this study was to test two hypotheses: 1) whether PTS in the medial and lateral compartments would be correlated with sagittal alignment under WB conditions; and 2) that PTS would be greater in the flexion contracture group than in the non-flexion contracture group for varus osteoarthritic knees.

## Materials and methods

The Institutional Review Board of Niigata University approved this study protocol. Approval number is 2351. All subjects permitted to use the data and provided their written or verbal informed consent at the time of the explanation for their surgery. The inclusion criteria for advanced knee osteoarthritis (OA) were subjects with advanced primary varus knee OA for the indication of primary total knee arthroplasty (TKA), unicompartmental knee arthroplasty (UKA), and high tibial osteotomy (HTO), which provided all of the necessary data for the 3D lower extremity alignment assessment system on biplanar long-leg radiographs, using 3D-to-2D image registration techniques [10, 11, 16, 17, 19–21]. Exclusion criteria were valgus OA, secondary OA following trauma, or other diseases. The knees were classified as grades 3–4, according to the Kellgren–Lawrence (K–L) classification, using X-rays [25]. Finally, we evaluated total 140 limbs of 106 subjects with grade 3–4 knee OA (107 limbs in 81 females and 33 limbs in 25 males; mean age, 75.1 years; age range, 62–87 years).

**Fig 2:**
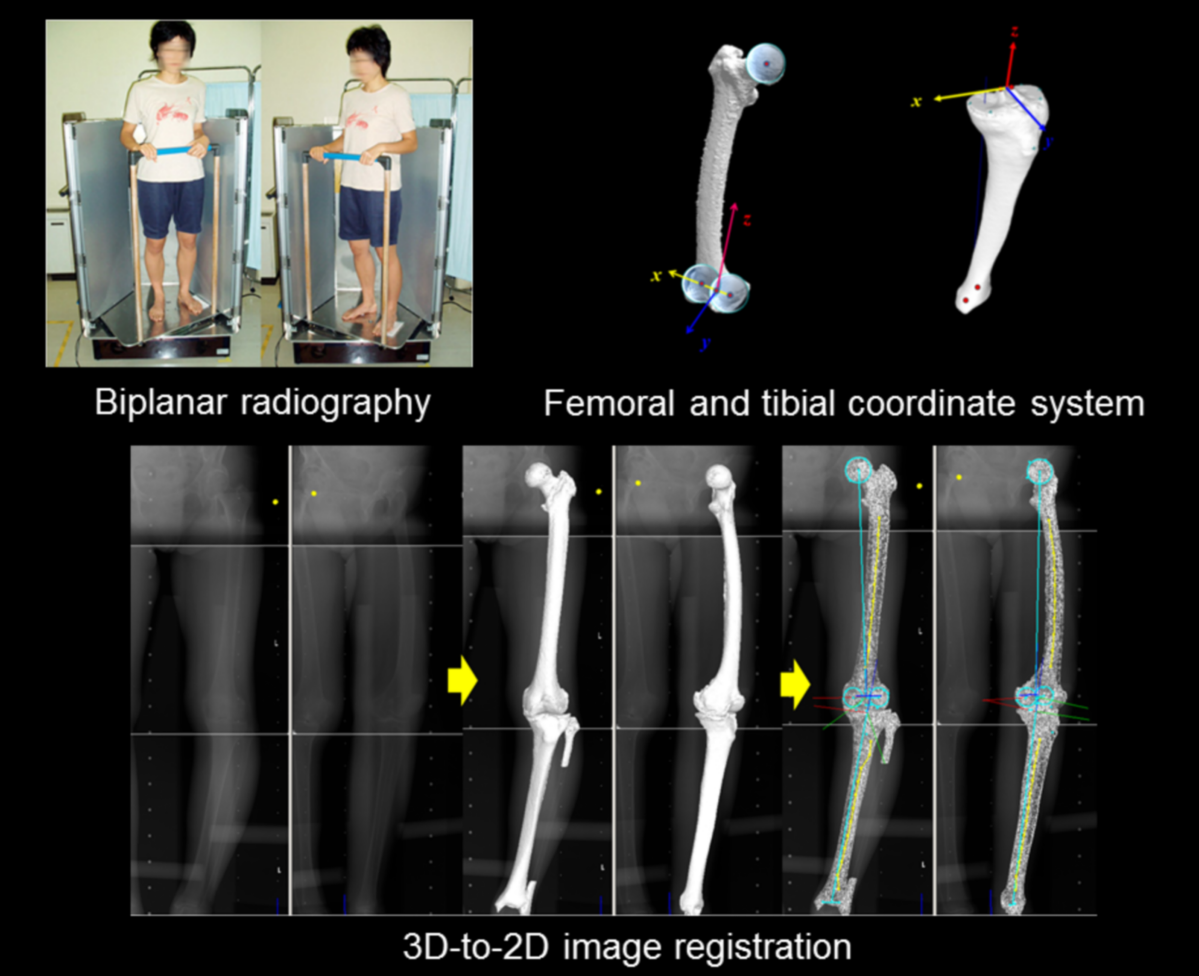
The biplanar radiography images of all the lower leg extremities under WB conditions. The 3D-to-2D image registration technique enabled the projection of the 3D digital bone models from the CT data, incorporating the anatomical coordinate system and the information of the morphology and alignment, onto biplanar radiography images.

In the 3D lower extremity alignment assessment system, firstly, a digital 3D model of the femur and tibia was reconstructed from computed tomography (CT) data (SOMATOM Sensation 16, Siemens Inc., Munich, Germany) using 3D visualization and modeling software (ZedView, LEXI Inc., Tokyo, Japan). And, the anatomical coordinate system was established according to the definition provided by Sato et al. [19] (Fig 2). For the femoral coordinate system, the geometric center axis (a line connecting the centers of spheres representing the medial and lateral posterior femoral condyles) was defined as the femoral x-axis (positive right). The origin of the coordinate system was defined as the midpoint between the centers of these posterior condylar spheres. The femoral y-axis was defined as a perpendicular line to the plane formed by the center of the femoral head and two centers of the posterior condylar spheres (positive anteriorly). The femoral z-axis (positive superiorly) was defined as the cross-product of the x-axis and y-axis (Fig 2). For the tibia, the z-axis was defined by a line connecting the midpoint of the tibial eminences and those of the medial and lateral top of the talar dome (positive superiorly). The tibial y-axis (positive anteriorly) was defined as the line perpendicular to the z-axis from the mediolateral center of the tibial insertion of the posterior cruciate ligament. The tibial x-axis was defined as the cross-product of the z-axis and y-axis (positive right) (Fig 2).

As the next procedure, the biplanar radiography images of all the lower leg extremities under WB conditions were obtained. The 3D-to-2D image registration technique enabled the projection of the 3D digital bone models from the CT data, incorporating the anatomical coordinate system and the information of the morphology and alignment, onto biplanar radiography images [10, 11, 16, 17, 19–21] (Fig 2). Thus, the accurate relative position (alignment) between the femur and tibia and morphology in the 3D space was automatically obtained following image-matching. The system accuracy was established as follows: three spherical markers were attached to each sawbone of the femur and tibia in order to define the local coordinate system. Outlines of the 3D bone models were projected onto extracted contours of each bone of the femur and tibia in the frontal and oblique radiography images. The 3D position of each model was recovered by minimizing the difference between the projected outline and contour. The accuracy was determined as the median values of the absolute error in estimating relative positions within 0.5 mm and 0.6° from the femur to tibia [11].

**Fig 3:**
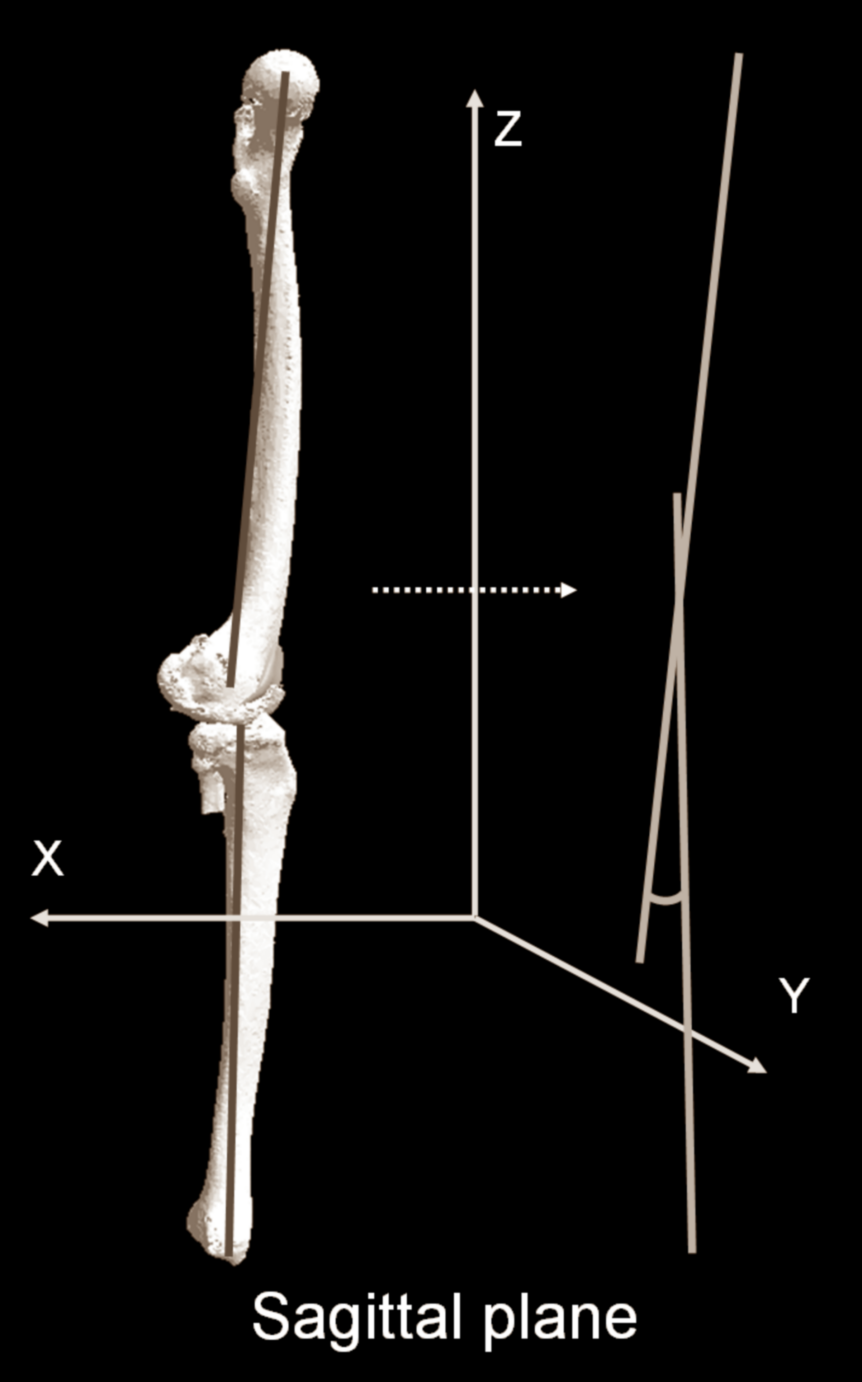
Schematic diagram showing sagittal alignment in 3D space [10].

The evaluation parameters were as follows: 1) 3D mechanical flexion angle (3DMFA) as sagittal alignment, 2) passing point in the WB line (PP) in the anteroposterior (AP) direction, and 3) medial and lateral PTS. 3DMFA, which was the extension–flexion angle at the maximum knee extension, was determined according to the definition reported by Ariumi et al. [10] (Fig 3). 3D functional axis of the femur (3DFA-f) was defined as a line connecting the center of the femoral head and the midpoint of the spheres that represented the medial and lateral posterior femoral condyles. 3D functional axis of the tibia (3DFA-t) was defined as a line connecting the midpoint of the eminences of the medial and lateral tibial spines and the center of the ankle joint. 3DMFA was determined as the angle between 3DFA-f and 3DFA-t projected onto the sagittal plane of the femoral coordinate system [10] (Fig 3). The subjects with 3DMFA >5° was defined as flexion contracture group (FC group: 93 limbs; mean age, 75.0 years; age range, 62–86 years) and those with 3DMFA <5° was defined as non-flexion contracture group (NFC group: 47 limbs; mean age, 75.1 years; age range, 62–87 years).

**Fig 4:**
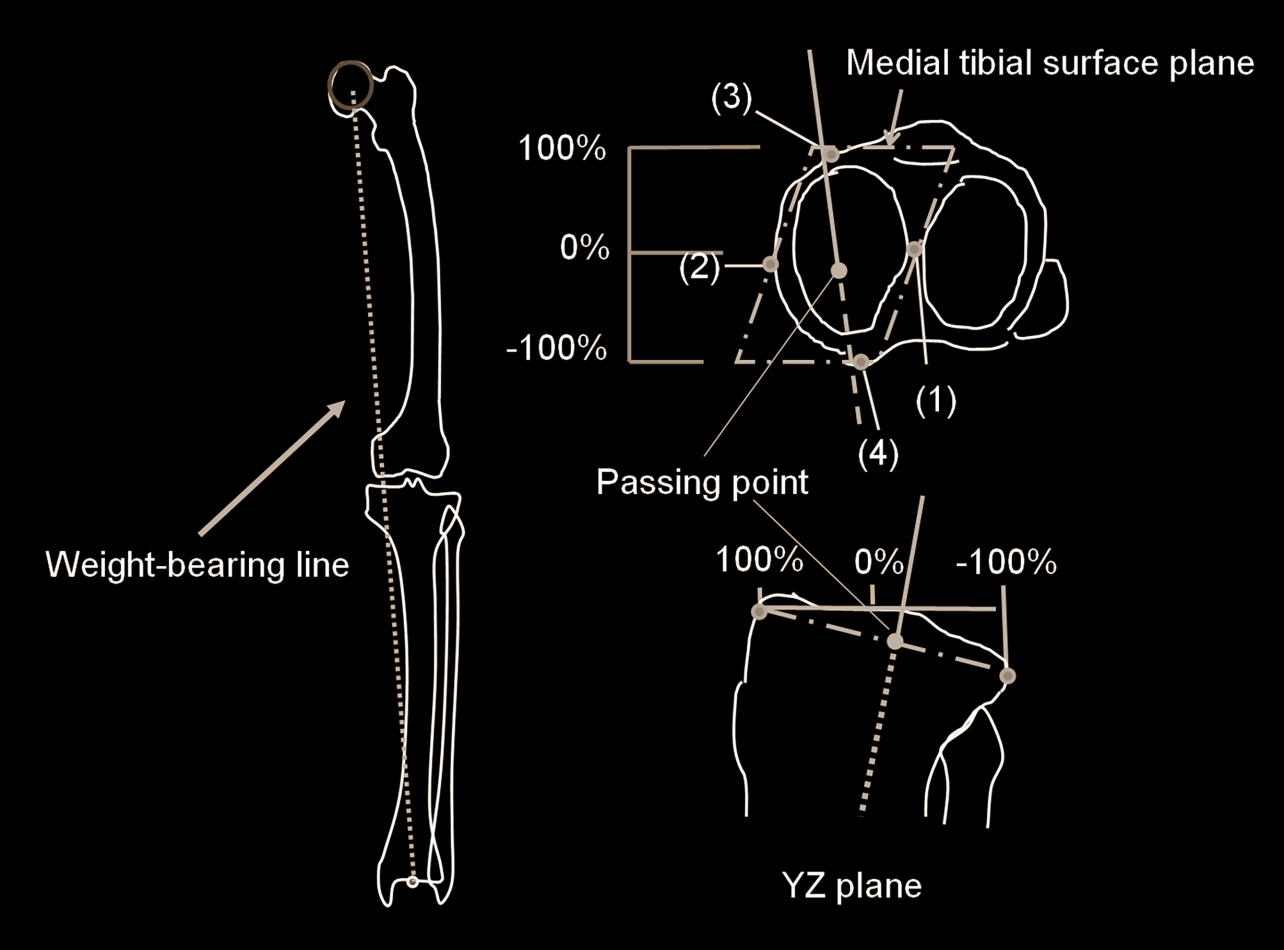
Schematic diagram showing the passing point of the weight-bearing line.

PP in the AP direction was defined as follows: PP, connecting the femoral head with the center of the ankle joint, was the location on the tibial surface plane defined by these four points: 1) the origin of the tibial coordinate system, 2) the medial-most point on the tibial surface, 3) the anterior-most point on the medial tibial surface, and 4) the posterior-most point on the medial tibial surface in the 3D space (Fig 4). PP in the tibial coordinate system was described by percent indication, which demonstrated that 0% meant the center between the anterior- and posterior-most point, +100% meant the anterior-most point, and −100% meant the posterior-most point on the medial tibial suface in the 3D space (Fig 4). The minus percent indication showed the posterior location from the center between the anterior- and posterior-most points.

**Fig 5:**
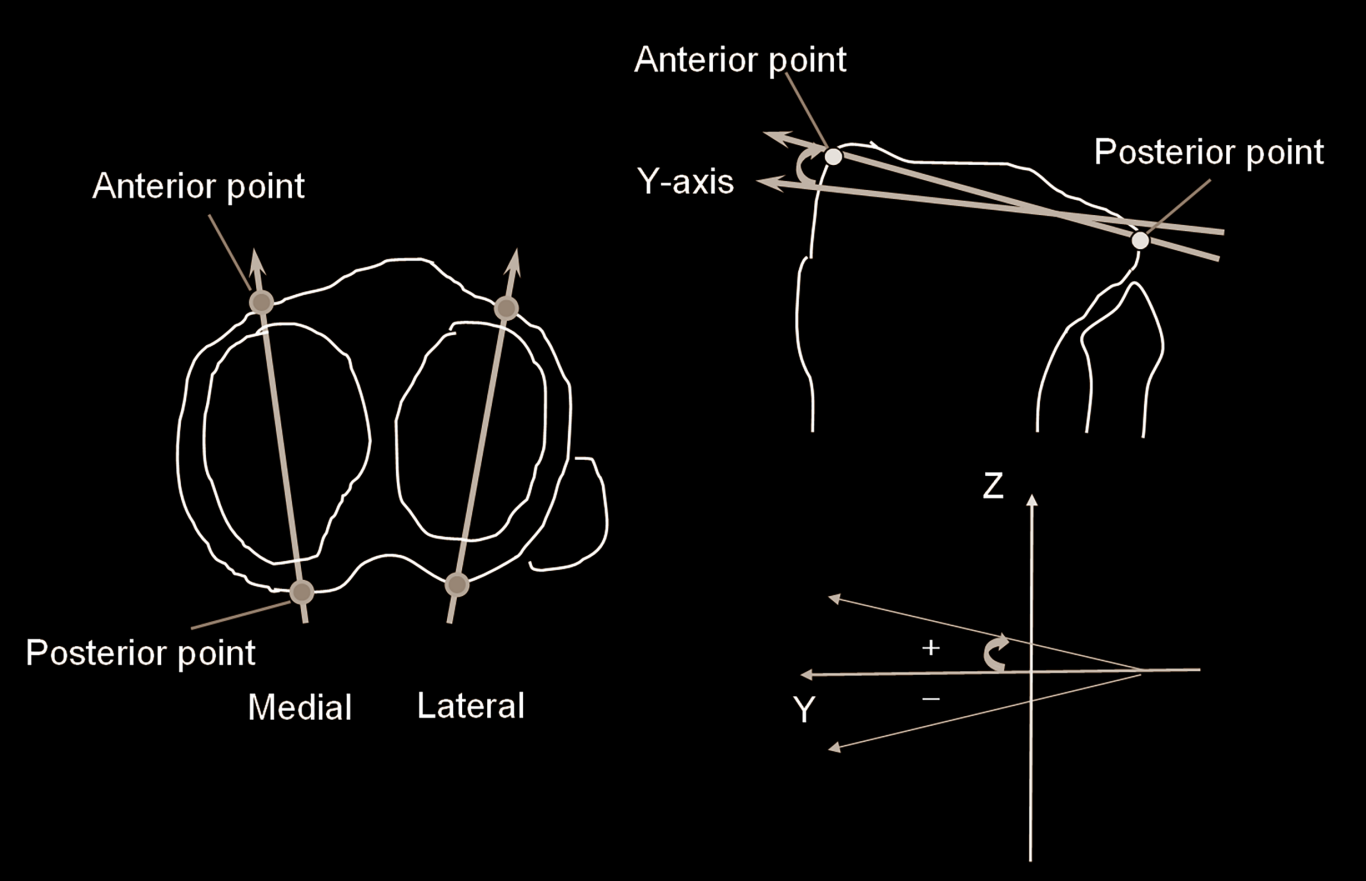
Schematic diagram showing posterior tibial slope.

Medial and lateral PTS were defined as the angle between the *y*-axis in the tibial coordinate system and the line connecting the anterior-most point to the posterior- most point on the medial and lateral tibial joint surface, projected onto the sagittal plane (*yz*) in the tibial coordinate system (Fig 5).

### Statistical analysis

Differences in the parameters were statistically analyzed using the Mann–Whitney U test. The correlation between each parameter was evaluated using the Spearman’s rank-order correlation. The probability level accepted for statistical significance was set at *p* < 0.05 (Version 21; SPSS, Inc., Chicago, IL, USA). The *post-hoc* power analysis showed high power in each parameter, respectively, when comparing the groups with or without flexion contracture (3DMFA: power = 1.000, *p* < 0.0001; PP: power = 1.000, *p* < 0.0001; Medial PTS: power = 0.803, *p* = 0.005; Lateral PTS: power = 0.780, *p* = 0.007).

## Results

When the FC group was compared with the NFC group, there were significant differences in 3DMFA (*p* < 0.0001) and PP (*p* < 0.0001) between the groups, respectively. Both PTS showed greater in the flexion contracture group than in the non-flexion contracture group, respectively (medial PTS: *p* = 0.006; lateral PTS: *p* = 0.006) (Table 1).

**Table 1:** Evaluation parameters.

	NFC group mean 95%CI		FC group Mean 95%CI		*p* value
Medial PTS (°)	6.7	5.5–7.9	9.2	8.1–10.4	0.006*
Lateral PTS (°)	7.2	4.8–9.5	10.9	9.4–12.5	0.006*
3D mechanical flexion angle (°)	0.0	-1.4–1.3	12.1	11.1–13.2	< 0.0001*
Passing point in WB line (%)	-8.3	-26.0–9.3	-163.2	-148.0 –-178.4	< 0.0001*

NFC = no flexion contracture; FC = flexion contracture; PTS = posterior tibial slope; 3D mechanical flexion angle: minus (-) means knee extension; Passing point in WB line: minus (-) means posterior direction; 95%CI = 95% confidence interval

*Significant difference = *p* < 0.05.

In the Spearman’s rank-order correlation, medial and lateral PTS were correlated with the 3DMFA and PP, respectively (medial PTS– 3DMFA: *p* = 0.001; medial PTS–PP: *p* < 0.0001; lateral PTS– 3DMFA: *p* < 0.0001; lateral PTS–PP: *p* = 0.002) (Table 2).

**Table 2:** Spearman’s rank-order correlation.

		Medial PTS	Lateral PTS
Medial PTS	CC	-	0.355
	*p* value	-	< 0.0001*
Lateral PTS	CC	0.355	-
	*p* value	< 0.0001*	-
3D mechanical flexion angle	CC	0.283	0.291
	*p* value	0.001*	< 0.0001*
Passing point in WB line	CC	0.361	0.265
	*p* value	< 0.0001*	0.002*

PTS = posterior tibial slope; CC = Correlation coefficient

*Significant difference = *p* < 0.05.

## Discussion

The investigation demonstrated that medial and lateral PTS were positively correlated to sagittal alignment under WB conditions. Furthermore, the flexion contracture group had larger PTS than the non-flexion contracture group. Clinically, these findings support a parallel mechanism for the sagittal plane in the tibial articular surface of osteoarthritic knees as the etiology for knee OA. Further, surgeons may be required to decrease the PTS angle in knee arthroplasty for patients to regain full extension in knees with preoperative fixed flexion contracture.

Preoperative flexion deformities require correction during knee arthroplasty. The clinical consequence of a fixed flexion deformity is that the quadriceps is continually forced to contract to avoid buckling. This exertion leads to a greater energy expenditure and results in fatigue [26–29]. Evidently, residual flexion contractures following knee arthroplasty have been associated with reduced functional scores and poor outcomes. Patients with the cruciate retaining insert and residual flexion contracture had increased pain and lower function scores [26, 30]. Surgical procedures play a key role in the potential for residual flexion contracture. While a number of factors, (e.g., insert and implant selection, gap balance, component position, alignment, release of a posterior cruciate ligament), are complicatedly connected for the improvement of the preoperative flexion contracture, a decrease in the PTS of the tibial cut will aid in the correction, since every degree of posterior slope equals a degree of residual flexion contracture [26]. The current study proved that PTS was more exaggerated in the flexion contracture group than in the non-flexion contracture group for the medial and lateral compartments of the tibial articular surface. The algorithm for correcting a fixed flexion deformity begins with the preoperative recognition of the problem. Preoperative PTS, particularly in the lateral compartment of varus osteoarthritic knees, is an important reference for determining the cutting angle on the tibia because the lateral compartment is generally in near normal condition. If a large PTS is not recognized preoperatively, an even steeper postoperative PTS can be produced and unintentionally cause residual flexion contracture. Therefore, using careful preoperative planning for knees with fixed flexion contracture, a decrease in the PTS angle via tibial cutting may be necessary to improvement.

PTS was correlated with the sagittal alignment under WB conditions, and PTS was greater in the flexion contracture group than in the non-flexion contracture group. It is assumed that PTS and sagittal alignment have a mutual influence on each other. When humans stand and experience gravitational force, the articular surface of the tibia is required to be aligned parallel to the ground for efficient load bearing (parallel mechanism), and a large PTS will be compensated by sagittal alignment [2] (Fig 1). We observed that subjects with a large PTS might stand with a slight knee flexion. It is possible that this stance is a comfortable standing position as the proximal tibial articular surface is aligned parallel to the ground by the bend in the knee.

The distribution of weight is important. The current study demonstrated that a greater PTS and increased compensation in sagittal alignment were associated with a more posterior WB line through the tibial proximal articular surface. A more posterior tibiofemoral contact point likely ensures increased contact pressure on the posterior part of the knee joint. In turn, during resting positions, this pressure can possibly cause a gradual change in the articular surface over time leading to increased PTS. This biomechanical negative cycle assumingly contributes to the progression of advanced knee OA. Furthermore, the etiology of a fixed flexion deformity is multifactorial. In addition to PTS, posterior capsular contracture, bony impingement, ligament contracture, and hamstring shortening contribute to the inability to completely straighten the knee [26]. These other factors can cause flexion contracture deterioration, and the sagittal alignment under WB conditions can worsen (increased knee flexion), thereby leading to greater PTS. Thus, the perpetual negative cycle of knee OA is further accelerated.

Limitations of this study include: 1) a relatively smaller number of male subjects and 2) non-inclusion of a moderate OA group (K–L grade 2), and 3) radiation exposure due to CT scanning.

## Conclusions

Medial and lateral PTS were positively correlated to sagittal alignment under WB conditions. Furthermore, the flexion contracture group had larger PTS than the non-flexion contracture group. Clinically, these findings support a parallel mechanism for the sagittal plane in the tibial articular surface of osteoarthritic knees as the etiology for knee OA. Surgeons may be required to decrease the PTS angle in knee arthroplasty for patients to regain full extension in knees with preoperative fixed flexion contracture.

## Supporting information

S1 File Data in this study. (XLSX)
